# Blunt chest trauma in older vs. Non-elderly adults: outcomes and management

**DOI:** 10.3389/fsurg.2026.1877092

**Published:** 2026-06-19

**Authors:** Ashraf F. Hefny, Nirmin A. Mansour, Fatima Aldhaheri, Fatima Alameri, Mohamed A. Hefny, Sherif A. Fathi, Taoufik Zoubeidi

**Affiliations:** 1 Department of Surgery, College of Medicine and Health Sciences, UAE University, Al Ain, United Arab Emirates; 2 Department of Surgery, Al Ain Hospital, Al Ain, United Arab Emirates; 3 Department of Family Medicine, Ambulatory Health Services, SEHA, Abu Dhabi, United Arab Emirates; 4 Department of Surgery, Faculty of Medicine, Ain Shams University, Cairo, Egypt; 5 Department of Surgery, Faculty of Medicine, October 6th University, Cairo, Egypt; 6 Department of Statistics, United Arab Emirates University, Al Ain, United Arab Emirates

**Keywords:** blunt trauma, chest, elderly, fall, road traffic collisions

## Abstract

**Background:**

Blunt chest trauma (BCT) substantially contributes to morbidity and mortality globally, especially in elderly adults. Most studies on BCT in older adults were conducted in Western countries, not reflecting regional differences. The study aimed to examine the incidence, injury patterns, management, and outcomes of BCT in older adults in the United Arab Emirates (UAE).

**Patient and methods:**

Data of patients aged ≥60 years with BCT admitted to a community-based hospital in 2014–2020 were retrospectively analyzed, including age, sex, nationality, mechanism of injury, vital signs, associated injuries, severity scales, hospitalization length, intensive care unit admission, ICU length of stay, management, and outcomes.

**Results:**

Of 861 patients assessed, 73 (8.5%) were aged ≥60 years. The most common mechanism of trauma was road traffic collisions (RTC; 51, 69.9%), followed by falling from height ≤1 meter (10, 13.7%). The most common thoracic injury was multiple rib fractures (25, 34.2%), whereas spine injury was the most common extrathoracic injury (25, 34.2%). Pulmonary contusion was significantly less frequent in older patients compared to younger adults with BCT (*P* = 0.004). Female sex and UAE national patients were significantly more frequent among older adults compared to younger adults with BCT (*P* < 0.001 and *P* = 0.002). Falls from a height of ≤1 meter were also significantly more common in older patients.

**Conclusions:**

Despite reduced occupational exposure and lower engagement in high-risk activities, RTC remain a major cause of BCT in elderly populations. Although many falls are low-energy events, their high incidence makes them a frequent contributor to the overall BCT burden in the elderly.

## Introduction

Blunt chest trauma (BCT) is a substantial contributor to morbidity and mortality among trauma patients, accounting for many hospital admissions and emergency interventions globally. Although road traffic collisions (RTC) remain the most common cause of BCT, mechanisms such as falls (particularly in older adults) are increasingly becoming prominent due to demographic shifts toward an aging population (1, 2).

Although definitions vary across trauma literature, patients aged ≥60 years were classified as elderly in this study in line with the World Health Organization (WHO) definition of older persons (2). Older adults are vulnerable in trauma care due to an age-related physiological decline, reduced cardiopulmonary reserve, higher incidence of comorbidities, and altered pharmacodynamics (2, 3). These changes increase the susceptibility to injury from seemingly low-energy mechanisms, such as falls from standing height, and complicate recovery after an injury, leading to longer hospitalization, higher complication rates, and increased health care utilization (4). By 2030, one in six people will be aged ≥60 years globally, highlighting the urgent necessity to better understand trauma patterns and outcomes in this demographic (5).

BCT in older adults presents unique challenges. For instance, rib fractures occur more frequently in this population and are associated with higher risks of pneumonia, prolonged ventilation, and mortality, even given the modest Injury Severity Score (ISS). Injuries may often be underestimated due to subtle clinical signs and atypical presentations in the elderly (6). Moreover, pre-existing conditions, such as osteoporosis and chronic cardiopulmonary disease, may exacerbate the severity of even minor thoracic trauma.

Despite the growing number of older trauma patients, the literature specifically addressing BCT in the elderly remains limited, particularly in the context of the Middle East and community hospital settings. Most available studies were conducted in major trauma centers in Western countries, which may not reflect the regional differences in injury mechanisms, healthcare access, or patient demographics.

Therefore, this study aimed to fill this knowledge gap by examining the incidence, injury patterns, management, and outcomes of BCT in older adults admitted to a community-based hospital in United Arab Emirates (UAE), a member state of the Gulf Cooperation Council countries. The rapid economic expansion of the UAE, similar to other high-income and fast-developing countries in the Middle East, has led to a substantial influx of young foreign laborers engaged in large-scale infrastructure projects. Currently, non-UAE nationals account for approximately 88%–89% according to recent data from the UAE Federal Competitiveness and Statistics Centre, with most expatriates returning to their countries of origin at retirement age (7).

We aimed to identify age-related trends that can inform clinical decision-making and guide the development of targeted prevention strategies by comparing geriatric patients to their younger counterparts. Ultimately, understanding these differences is critical for optimizing trauma care, improving outcomes, and shaping policy in trauma systems that are increasingly encountering older adults.

## Materials and methods

Older adult patients (≥60 years) with BCT who were admitted to Al Ain Hospital between December 2014 and January 2020 were retrospectively analyzed using the trauma registry. The latter included patients of all ages who were admitted to the hospital or died in the Emergency Department during the study period, excluding patients who died at the scene or before reaching the hospital, as well as those discharged from the Emergency Department. Patients aged <18 years were excluded from study. Most of the missing information from the trauma registry was collected from the electronic medical files of the patients.

The collected data included age, sex, nationality, mechanism of injury, vital signs on admission, associated injuries, Glasgow Coma Score (GCS), Injury Severity Score (ISS), New Injury Severity Score (NISS), Abbreviated Injury Scale (AIS), length of hospital stay (LOS), intensive care unit (ICU) admission, ICU length of stay, management, and outcomes.

Predefined comorbidities (including hypertension, diabetes mellitus, and other chronic conditions) were systematically collected from trauma registry records. These conditions were considered present if documented in the patient's medical history or if the patient was receiving disease-specific treatment at admission. All comorbidities were categorized as binary variables (present/absent) for statistical analysis. The severity of injuries and outcomes were compared between older (≥60 years) and younger adult patients (18–59 years).

All statistical analyses were performed using IBM SPSS Statistics software (version 28). Data were reported as median (IQR) or numbers (%). For continuous or ordinal data, the Mann–Whitney U test was used to compare two independent groups. Differences between categorical variables were tested using Pearson's chi-square test and Fisher's exact test, with the latter used in two-by-two tables when the assumptions of the chi-square test were not satisfied. Since the assumptions of normality and homogeneity of variances for were not met for all variables, the Welch's test, which is robust to these violations, was used to assess differences in means across the groups. Provided the Welch's test was not applicable, the Kruskal–Wallis test was employed to compare medians. Additionally, the female 60 + group was excluded from analysis when it contained <2 cases. For significant overall differences, *post hoc* pairwise comparisons were conducted using the Games–Howell test. Statistical significance was established at a 5% significance level.

A penalized likelihood logistic regression (Firth's bias-reduced logistic regression) of ICU admission vs. age group (18–59, 60 + years), gender, pulse per minute, systolic blood pressure, respiratory rate, GCS, ISS, NISS, AIS Thorax, hospital LOS and the two-way interactions of age group and gender with all the remaining predictors was considered. Firth's logistic regression was use instead of the regular logistic regression because there were indications of complete or quasi complete separations in the data of these variables. The standard errors of the coefficients in the regular logistic regression were highly inflated.

Informed consent was obtained from all the patients who were admitted to the hospital or from their guardians. This study was approved by Tawam Human Research Ethics Committee, Abu Dhabi, UAE, approved this study (T-HREC ref. MF2058-2023-952).

## Results

During the study, 861 patients were admitted to our institution with BCT. Of these, 73 (8.5%) patients were aged ≥60 years. Table 1 shows the basic characteristics of those patients. The UAE nationals were the most frequently admitted patients (22 patients; 30.1%), followed by Sudani nationality (9 patients; 12.3%). The trauma occurred on the city streets in 35 (47.9%) patients and at home in 26 (35.6%) patients. The most common mechanism of trauma was RTC (51 patients; 69.9%), followed by falling from height ≤1 meter (10 patients; 13.7%) and falling from >1 meter (9 patients; 12.3%). Thirty-eight (52.1%) patients were involved in a care-care collision, of whom 26 (68.4%) were drivers. BCT was work-related in only 2 (2.7%) patients. Nineteen (26%) patients had comorbidities such as diabetes mellitus, hypertension, and other chronic medical diseases.

**Table 1:** Baseline characteristics in older adult patients who sustained BCT (*n* = 73).

Key descriptive characteristics	
Demographics and vital signs	
Male, *n* (%)	52 (71.2%)
Female, *n* (%)	21 (28.8%)
Age (years) median (IQR)	68 (11)
Systolic blood pressure mmHg, median (IQR)	138 (24)
Pulse beats/min, median (IQR)	88 (19)
Respiratory rate breath/min, median (IQR)	18 (2)
GCS, median (IQR)	15 (0)
^a^Common thoracic injuries *n* (%)	
Multiple rib fractures	25 (34.2)
Pulmonary contusion	14 (19.2%)
Pneumothorax	11 (15.1%)
Surgical emphysema	5 (6.8%)
Sternum fractures	4 (5.5%)
Hemothorax	4 (5.5%)
Great vessels injury	1 (1.4%)
^a^Extrathoracic injuries *n* (%)	
Spine injury	25 (34.2%)
Face injury	24 (32.9%)
Lower extremity injury	24 (32.9%)
Upper extremity injury	24 (32.9%)
Abdominal injury	18 (24.7%)
Head injury	13 (17.8%)
Neck injury	11 (15.1%)
Injury Severity Scores, median (IQR)	
ISS	5 (8)
NISS	7 (10)
Hospital length of stay (days), median (IQR)	3 (5)

a The percentage is more than 100% because some patients had more than one injury.

The most common thoracic injury was multiple rib fractures (25 patients; 34.2%), whereas spine injury was the most common extrathoracic injury (25 patients; 34.2%; Table 1). During treatment, 3 (4.1%) patients received blood transfusions, 4 (5.5%) patients had chest tube insertion, and 6 (8.2%) patients were admitted to an ICU, of whom 4 (5.5%) were intubated.

Pulmonary contusion was significantly less frequent in older patients compared to younger adults. Moreover, pneumothorax tended to occur less frequently in older adults. Additionally, head and upper limb injuries tended to occur more commonly in younger adult patients (Table 2).

**Table 2:** Thoracic and extra-thoracic injuries in older adults’ patients (*n* = 73) compared to other younger BCT patients (*n* = 788).

Injury	Older adults *n* (%)	Younger patients *n* (%)	Total	*p*-value^a^
Pulmonary contusion	14 (19.2)	283 (35.9)	297 (34.7)	**0.004**
Hemothorax	4 (5.5)	65 (8.2)	69 (8)	0.404
Pneumothorax	11 (15.1)	193 (24.5)	204 (23.7)	0.07
Multiple rib fractures	25 (34.2)	228 (28.9)	253 (29.4)	0.340
Surgical emphysema	5 (6.8)	48 (6.1)	53 (6.2)	0.797
Sternum fracture	4 (5.5)	32 (4.1)	36 (4.2)	0.562
Great vessels injury	1 (1.4)	5 (0.6)	6 (0.7)	0.470
Single rib fracture	2 (2.7)	69 (8.8)	71 (8.2)	0.077
Head	13 (17.8)	231 (29.3)	255 (29.6)	0.057
Face	24 (32.9)	260 (33)	284 (28.5)	0.524
Neck injury	11 (15.1)	123 (15.6)	134 (15.6)	0.903
Abdomen injury	18 (24.7)	200 (25.4)	218 (25.3)	0.892
Spine injury	25 (34.2)	341 (39.8)	366 (42.5)	0.136
Upper extremity	24 (32.9)	349 (44.3)	373 (43.3)	0.060
Lower extremity	24 (32.9)	340 (43.1)	364 (42.3)	0.089

a Pearson's chi-square test or Fisher's Exact Test.

*P* value <0.05 is considered significant (bold value).

Older adults had a significantly higher proportion of female and UAE national patients than younger adults with BCT (*P* < 0.001 and *P* = 0.002). Home and street trauma was significantly more and less frequent in older adults, respectively (*P* < 0.001 and *P* = 0.025, respectively). Falls from a height of ≤1 meter were significantly more common in older patients compared to young adults. Furthermore, work-related injuries were significantly less frequent in older adults (*P* < 0.001). Older adults had significantly more comorbidities compared to younger patients (Table 3).

**Table 3:** Demography, mechanism, place of injury, and comorbidities in older adult patients (*n* = 73) compared to other younger BCT patients (*n* = 788).

Variable	Older adults (*n* = 73)	Younger patients (*n* = 788)	Total (*n* = 861)	*P*-value^a^
Demography				
Sex: Female	21 (28.8%)	92 (11.7%)	113 (13.1%)	**<0** **.** **001**
Male	52 (71.2%)	696 (88.3%)	748 (86.9%)	
UAE Nationals	22 (30.1%)	124 (15.7%)	146 (17%)	**0**.**002**
Mechanism				
RTC	51 (69.9%)	515 (65.4%)	566 (65.7%)	0.498
Fall from > one meter	7 (9.6%)	134 (17%)	141 (16.4%)	0.096
Fall from < one meter	10 (13.7%)	44 (5.6%)	54 (6.3%)	**0**.**007**
Place of injury				
Street	35 (47.9%)	479 (60.8%)	514 (59.7%)	**0**.**025**
Home	26 (35.6%)	61 (7.7%)	87 (10.1%)	**<0**.**001**
Work related injury	2 (2.7%)	135 (17.1%)	137 (15.9%)	**<0**.**001**
Comorbidity	19 (26%)	47 (5.9%)	66 (7.7%)	**<0**.**001**

a Pearson's chi-square test or Fisher's Exact Test.

*P* value <0.05 is considered significant (bold value).

RTC, road traffic collusions.

No significant differences in vital signs on admission and AIS of the thorax were identified between older adults and younger adults. However, NISS was significantly lower in older adults. The ISS tended to be lower in older adults compared to younger adults (Table 4). The two groups did not show significant differences for operative and non-operative management (Table 5).

**Table 4:** Vital signs and injury severity scores in older adults’ patients (*n* = 73) compared to other younger BCT patients (*n* = 788).

Variable	Statistics	Older adults	Younger patients	*P*-value^a^
		(*n* = 73)	(*n* = 788)	
Pulse (bpm)	Median	89	86	0.692
	Minimum	58	0	
	Maximum	151	180	
	IQR	19	20	
Systolic BP (mmHg)	Median	138	134	0.148
	Minimum	89	0	
	Maximum	210	203	
	IQR	29	25	
RR (per minute)	Median	18	18	0.097
	Minimum	16	0	
	Maximum	28	45	
	IQR	2	2	
GCS	Median	15	15	0.867
	Minimum	3	3	
	Maximum	15	15	
	IQR	0	0	
ISS	Median	5	8	0.065
	Minimum	1	1	
	Maximum	26	75	
	IQR	8	9	
NISS	Median	7	9	**0.031**
	Minimum	1	1	
	Maximum	29	75	
	IQR	10	13	
AIS Thorax	Median	2	2	0.925
	Minimum	1	1	
	Maximum	4	5	
	IQR	2	2	
Hospital LOS	Median	2.5	2	0.511
	Minimum	1	1	
	Maximum	24	74	
	IQR	4	4	

GCS, glasgow coma scale; ISS, injury severity score; NISS, new injury severity score; ICU, intensive care unit; RR, respiratory rate; BP, blood pressure; AIS, abbreviated injury scale; LOS, length of stay.

a Mann–Whitney Test.

a *P* value <0.05 is considered significant (bold value).

**Table 5:** Cardiac enzymes levels and management in older adults’ patients (*n* = 73) compared to other younger BCT patients (*n* = 788).

Variable	Older adults (*n* = 73)	Younger patients (*n* = 788)	Total (*n* = 861)	*P*-value^a^
Cardiac enzymes				
High troponin	4 (5.5%)	34 (4.3%)	38 (4.4%)	0.752
High creatine kinase	1 (1.4%)	34 (4.3%)	35 (4.1%)	1.0
High CK-MB	3 (4.1%)	24 (3%)	27 (3.1%)	0.717
Management				
ICU admission	6 (8.2%)	121 (15.4%)	127 (14.8%)	0.1
Blood transfusion	3 (4.1%)	84 (10.7%)	87 (10.1%)	0.1
Chest tube insertion	4 (5.5%)	52 (6.6%)	56 (6.5%)	0.475
Conservative management	54 (74%)	573 (72.7%)	627 (72.8%)	0.817
Thoracic operations	2 (2.7%)	29 (3.7%)	31 (3.6%)	0.523

ICU, intensive care unit; CK-MB, creatine kinase-myocardial band.

*P* value <0.05 is considered significant.

a Pearson's chi-square test or Fisher's Exact Test.

ICU admission does not seem to be significantly different between the elderly (60 + years) and the young (18–59 years) and between males and females (*p*-values > 0.1). The likelihood of ICU admission is significantly affected by GCS, ISS, and respiratory rate (Table 6).

**Table 6:** Multiple logistic regression of ICU admission vs. GCS, ISS, RR while controlling for Age group and Gender. A backward elimination procedure was used using a penalized likelihood test.

Variable	Coefficient	SE of coefficient	OR	95% CI for Odd Ratio		*p*-value^a^
				lower	Upper	
(Intercept)	−2.253	1.150	0.105	0.011	1.098	0.059
Age group	−0.382	0.499	0.682	0.228	1.705	0.434
Gender	0.336	0.374	1.399	0.642	2.848	0.384
ISS	0.101	0.015	1.106	1.075	1.139	**<0** **.** **001**
RR	0.155	0.028	1.168	1.105	1.238	**<0**.**001**
GCS	−0.284	0.053	0.753	0.673	0.833	**<0**.**001**

ICU, intensive care unit; Age Group (18–59, 60 + yrs), GCS, glasgow coma scale; ISS, injury severity score; RR, respiratory rate.

a Profile penalized log-likelihood.

*P* value <0.05 is considered significant (bold value).

**Figure 1:**
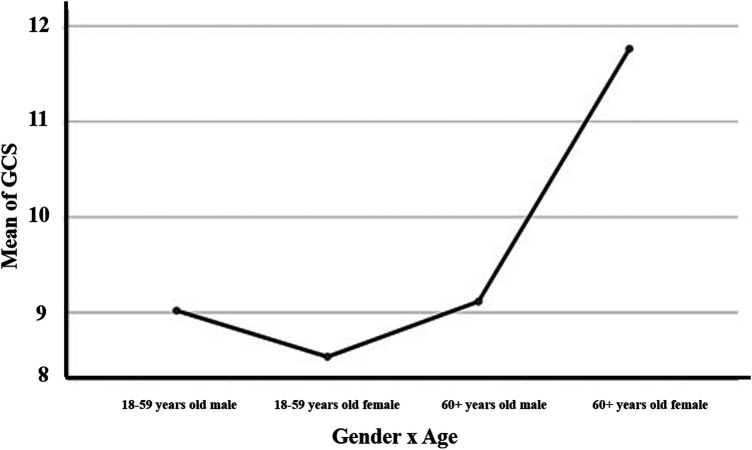
Comparative analysis of GCS mean across four sex-age groups: males aged 18–59 years, females aged 18–59 years, males aged 60 years and above, and females aged 60 years and above.

**Figure 2:**
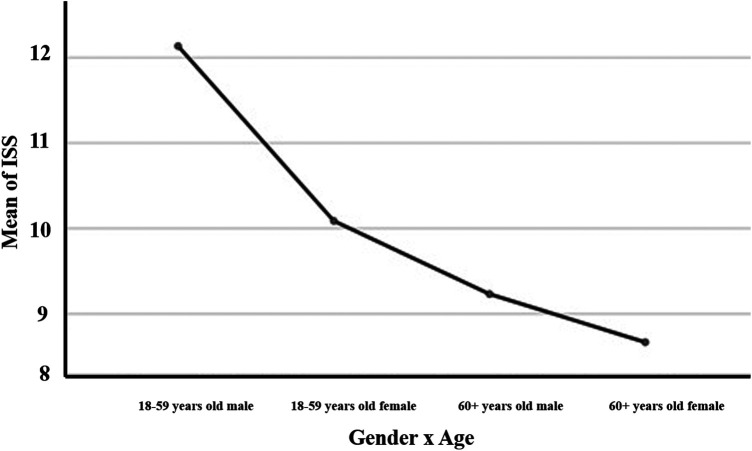
Comparative analysis of ISS mean across four sex-age groups: males aged 18–59 years, females aged 18–59 years, males aged 60 years and above, and females aged 60 years and above.

**Figure 3:**
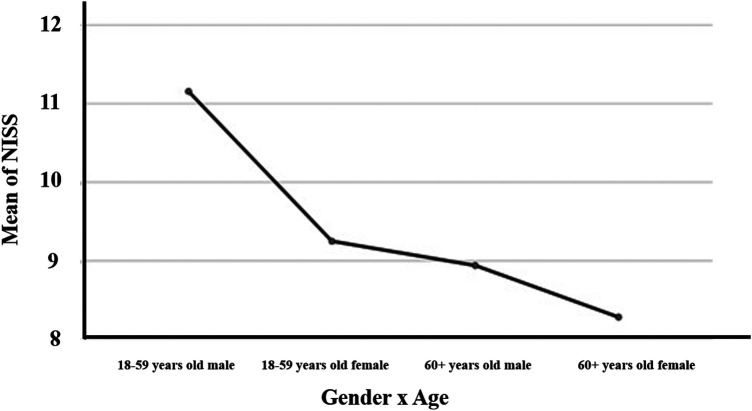
Comparative analysis of NISS mean across four sex-age groups: males aged 18–59 years, females aged 18–59 years, males aged 60 years and above, and females aged 60 years and above.

Table 7 shows the comparative analysis of quantitative variables across four sex-age groups: males aged 18–59 years, females aged 18–59 years, males aged ≥60 years, and females aged ≥60 years. Significant differences were observed in mean GCS scores, with female patients aged 18–59 years exhibiting lower values than females aged ≥60 years (*P* < 0.001; Figure 1). The ISS and NISS scores were significantly elevated among younger patients compared to older adults (both *P* < 0.05; Figures 2, 3).

**Table 7:** Comparative analysis of quantitative variables across four sex-age groups: males aged 18–59, females aged 18–59, males aged 60+, and females aged 60 + .

Dependent Variable	18–59 years old male			18–59 years old female			60 + years old male			60 + years old female			*p*-value^a^
	N	Mean (SD)	95% Ci for Mean	N	Mean (SD)	95% Ci for Mean	N	Mean (SD)	95% Ci for Mean	N	Mean (SD)	95% Ci for Mean	
Systolic BP	678	134.71 (22.63)	(133, 136.42)	88	133.41 (20.56)	(129.05, 137.77)	51	139.25 (23.02)	(132.78, 145.73)	21	137.33 (19.93)	(128.26, 146.4)	0.463
Pulse	695	88.04 (19.72)	(86.57, 89.51)	92	89.5 (17.25)	(85.93, 93.07)	52	88.38 (18.86)	(83.14, 93.63)	20	89.7 (16.4)	(82.02, 97.38)	0.874
Respiratory rate	688	19.46 (4.03)	(19.16, 19.77)	89	19.12 (3.4)	(18.41, 19.84)	52	19.48 (3.58)	(18.48, 20.48)	21	18.57 (2.68)	(17.35, 19.79)	0.444
GCS	693	14.4 (2.23)	(14.24, 14.57)	91	14.31 (2.5)	(13.79, 14.83)	52	14.42 (2.01)	(13.86, 14.98)	21	14.95 (0.22)	(14.85, 15.05)	**<0** **.** **001**
ICU LOS	107	8.97 (11.71)	(6.73, 11.22)	14	15.07 (13.06)	(7.53, 22.61)	5	16.4 (20.47)	(-9.01, 41.81)	NA	NA	NA	0.268
ISS	687	11.16 (9.54)	(10.44, 11.87)	88	9.25 (8.6)	(7.43, 11.07)	51	8.94 (7.83)	(6.74, 11.14)	21	8.29 (5.06)	(5.98, 10.59)	**0**.**016**
NISS	695	13.14 (10.73)	(12.34, 13.93)	91	11.09 (9.74)	(9.06, 13.12)	52	10.23 (8.48)	(7.87, 12.59)	21	9.67 (6.45)	(6.73, 12.6)	0.011
Hospital LOS	696	6.11 (8.83)	(5.45, 6.77)	92	5.9 (10.45)	(3.74, 8.07)	51	5.53 (8.03)	(3.27, 7.79)	21	4.43 (5.21)	(2.06, 6.8)	0.552
AIS head	203	2.11 (1.28)	(1.94, 2.29)	19	1.79 (0.79)	(1.41, 2.17)	13	1.85 (1.21)	(1.11, 2.58)	NA	NA	NA	0.262
AIS face	129	1.43 (0.66)	(1.31, 1.54)	16	1.25 (0.45)	(1.01, 1.49)	14	1.36 (0.5)	(1.07, 1.64)	4	1 (0)	(1, 1)	0.402^b^
AIS neck	65	1.05 (0.28)	(0.98, 1.11)	12	1 (0)	(1, 1)	5	1 (0)	(1, 1)	NA	NA	NA	0.767^b^
AIS thorax	696	2.06 (1.09)	(1.98, 2.14)	92	1.84 (1.06)	(1.62, 2.06)	52	2 (0.95)	(1.74, 2.26)	21	1.95 (1.07)	(1.46, 2.44)	0.314
AIS abdomen	165	1.56 (0.81)	(1.44, 1.69)	24	1.71 (0.96)	(1.31, 2.11)	8	1.38 (0.74)	(0.75, 2)	5	1.2 (0.45)	(0.64, 1.76)	0.327
AIS spine	216	1.98 (0.57)	(1.9, 2.06)	30	1.73 (0.58)	(1.52, 1.95)	12	1.92 (0.52)	(1.59, 2.24)	8	2.25 (0.46)	(1.86, 2.64)	0.086
AIS upper extremity	207	1.82 (0.56)	(1.74, 1.89)	27	1.85 (0.6)	(1.61, 2.09)	11	1.91 (0.7)	(1.44, 2.38)	5	1.6 (0.55)	(0.92, 2.28)	0.808
AIS lower extremity	183	1.84 (0.77)	(1.73, 1.95)	30	1.8 (0.85)	(1.48, 2.12)	11	1.82 (0.87)	(1.23, 2.41)	NA	NA	NA	0.934^b^

BP, blood pressure; GCS, glasgow coma scale; ICU, intensive care unit; LOS, length of stay; ISS, injury severity score; NISS, new injury severity score; RR, AIS, abbreviated injury scale.

a Welsh test.

b Kruskal–Wallis test.

*P* value <0.05 is considered significant (bold value).

Conservative management was adopted in 54 (74%) patients. Nineteen (26%) patients underwent surgery, with nonthoracic surgery in 17 (89.5%) cases.

No patients died in the older adult group compared to 17 (2.2%) patients in the other group of adult patients. LOS showed no significant difference between the two groups (Table 4).

## Discussion

Older adults (≥60 years) represent a rapidly growing population in the UAE, with estimates around 305,371 (3.2%) in 2020, which are projected to reach 2 million (nearly 20%) by 2050. This marked demographic shift toward the aging population creates new challenges for healthcare and social services (8).

Elderly patients accounted for about 8% of those admitted with BCT in the present study, indicating that older adults are overrepresented among BCT patients relative to their share of the general population. Similarly, a US study showed that while older adults constituted about 18% of the population, they accounted for approximately 30% of individuals with BCT (9).

Of elderly patients admitted with BCT, Emirati nationals represented almost one-third, which is significantly higher than in younger adults. This is likely because expatriates predominate the population of the UAE, as they tend to return to their home countries upon retirement or with declining health. Consequently, the pool of older expatriates in the UAE is small, making the proportion of Emirati nationals significantly higher among elderly trauma admissions (10).

Similar to other countries, older women with BCT outnumbered their male counterparts in the UAE. The current study showed that older patients with BCT were significantly more commonly female compared to younger adults. This could be partly attributable to the longer female life duration observed in the UAE (11).

The increasing national longevity reinforces the structural pattern in which women will comprise a larger segment of the aging population, which emphasizes the significance of sex-specific considerations in geriatric health planning and trauma prevention in the UAE (12).

RTC and work-related injuries occur significantly more frequently in younger adults compared with elderly patients. Moreover, as many elderly patients continue to use private or public transportation, RTC remains a leading cause of BCT in this age group despite reduced outdoor activities (13).

Most elderly patients who were involved in car-care collisions were drivers (68%). The anterior chest wall of a driver is exposed to direct impact and compression between the seatbelt restraint, steering wheel, or other interior parts of the car, predisposing them to rib fractures, pulmonary contusion, and other chest injuries (14).

Overall, roads are the most common location for BCT in adults. However, when focusing on elderly patients, home injuries, especially domestic falls, are significantly more commonly reported in older adults compared to younger adults (15).

Falls constitute another common cause of BCT in elderly populations. Older adults fell from standing height or ≤1 meter more frequently compared to their younger counterparts. Epidemiological studies indicated that unintentional ground-level falls account for the majority of trauma cases in older adults, mostly occurring at home or in familiar environments, such as bathrooms, stairs, or bedrooms. Such a low-energy traumatic event is especially frequent due to an age-related decrease in balance, muscle strength, and sensorimotor coordination (1). Although traditionally considered low energy, these falls can cause clinically significant injury, particularly in frail elderly individuals with comorbidities or reduced bone density.

Direct chest impact against rigid surfaces such as floors, furniture, or stairs can result in localized force transmission sufficient to cause chest wall injury, including rib fractures and soft tissue contusion (16, 17). The frequency of falls and patient's vulnerability highlight their importance as a common mechanism of BCT in the elderly (18).

Since the fall risk increases markedly with advancing age, the incidence of fall-related events will continue to rise as the population ages (19). Evidence-based interventions emphasize that modifying the home environment plays a critical role in reducing fall risk among older adults. Effective environmental modifications include eliminating household hazards (such as clutter and loose cords), improving lighting, securing or removing unstable rugs, and installing structural supports (e.g., grab bars, non-slip mats, and enhanced bathroom safety features). Additionally, promoting fall prevention exercises, such as regular daily physical activity, balance training, and gait strengthening exercises, significantly decreases the likelihood of falls among older adults (20).

Similar to another study, the current study showed that multiple rib fractures are common in the elderly with BCT. This reflects the age-related bone fragility and diminished chest wall capacity to absorb impact forces even from low-energy traumatic events (15).

In the present study, pulmonary contusion was significantly less frequent in older patients compared with young ones. The following two main factors likely explain this observation. First, age-related physical changes in the chest wall, including a stiffer, less compliant thoracic cage and more fragile ribs, tend to divert traumatic forces toward rib fractures rather than causing injury to the lung parenchyma. Second, higher energy trauma force in younger adults, such as RTC produce significant lung injury compared to older adult patients with low-energy trauma, such as ground-level falls. These findings are supported by previous studies, similarly, demonstrating that pulmonary contusions are more frequently observed in younger patients, whereas rib fractures are more prevalent in the elderly (21, 22).

The current study showed that spine injury was the most common extrathoracic injury in geriatric BCT. The age-related degenerative changes, osteoporosis, low bone mineral density, and reduced spinal flexibility increase the threshold for compression vertebral fractures and cervical injuries. Hence, elderly patients may sustain spinal injury from relatively low-energy traumatic events, including a fall from <1 meter, which might not produce spinal injury in younger individuals (23).

There was a trend toward a higher frequency of head and upper limb injuries among younger patients. Other reports did not demonstrate a statistically significant age-related trend in extrathoracic injuries compared to the younger population, despite clear differences in thoracic skeletal injury patterns. Thus, the apparent predominance of head and upper limb injuries among younger patients in our study may be influenced by the mechanism of injury (15).

The GCS is significantly associated with mortality among patients with BCT (24).

A previous large retrospective study showed that younger adults often present with lower GCS profiles compared with elderly patients, even with similar injury severity scores (25).

Although the current study did not find a significant difference between both elderly and younger men in terms of GCS, younger females had significantly lower GCS compared to elderly females. The lower GCS indicates severer head injury in younger females, which occur due to higher trauma energy in younger females compared to elderly.

This study showed that the ISS and NISS were lower in elderly patients as they frequently sustain low-energy traumatic events (e.g., falls from the ground level), resulting in injuries that may receive lower ISS and NISS scores. However, these scores underestimate the true injury severity in the elderly as they do not incorporate the physiological reserve, frailty, or comorbidities that result in worse outcomes for elderly patients compared to younger with the same ISS (26, 27).

The primary management strategy in elderly patients with BCT is nonsurgical treatment. The cornerstone of therapy for most older adult patients with rib fractures and other chest injuries is supportive management, including pain control, oxygen supply, respiratory care, physiotherapy, and close monitoring (28). This conservative approach is particularly advocated for stable injuries without flail segments or serious intrathoracic injuries that mandate operative intervention. This helps to minimize the risk of complications related to invasive procedures in a population with reduced physiological reserve. Furthermore, most patients in the current study who underwent surgery had extrathoracic associated injuries, particularly involving the extremities, abdomen, and the central nervous system. This finding is consistent with previously published trauma series reporting that approximately 45%–60% of elderly patients with BCT sustain significant extrathoracic injuries, many of which necessitate surgery (29).

Table 5 demonstrates comparable cardiac enzyme elevations and management strategies between older and younger patients with BCT, with no statistically significant differences observed. However, the relatively small sample size in the older adult group may have limited the statistical power to detect clinically relevant differences, particularly in variables such as ICU admission and blood transfusion requirements.

ICU admission appears to be primarily driven by clinical severity parameters rather than demographic characteristics. In the final multivariable model, neither age group nor gender demonstrated a significant association with ICU admission, whereas ISS, GCS, and respiratory rate were significant predictors. Specifically, increasing ISS and respiratory rate were associated with higher odds of ICU admission, while increasing GCS was associated with reduced odds. These findings robustly demonstrate that the decision for ICU admission is driven by injury severity and physiological derangement at presentation, rather than by patient age or sex which is similar to another study (30).

Elderly patients with BCT have substantially higher mortality and morbidity compared to younger adults with similar injury severity scores (18). However, no mortality was observed among elderly patients in this cohort. This finding should be interpreted with caution, as it may be attributed to the relatively small number of elderly patients, differences in injury mechanisms and potential selection bias, given that prehospital deaths were not captured within the trauma registry.

## Limitations

This study was conducted retrospectively at a single center and involved a relatively small elderly sample size, which might limit the generalizability of the findings. As with most retrospective designs, the key limitations of reliance on previously recorded information include risks of incomplete documentation, potential information bias, lack of adjustment for major confounders (comorbidities, frailty, and medications), and inadequate details on management strategies and long-term outcomes. To reduce these limitations, we implemented rigorous data verification, ensuring that all available variables were collected accurately and consistently, including manual review of missing details or unclear entries from the electronic medical records of the patients.

Some important data, such as alcohol abuse and seatbelt use, were not included in the trauma registry or medical records. The mortality rate might have been underestimated, as patients who died before reaching the hospital were not included in the trauma registry. Therefore, given its descriptive and hypothesis-generating nature, this study should be interpreted with caution, and further validation through larger, prospective, multicenter investigations is necessary.

## Conclusions

Road traffic collisions remain a leading cause of blunt chest trauma in the elderly. Low-level falls (≤1 meter), predominantly occurring at home, represent a substantial and preventable contributor. With the rapidly expanding elderly population in the UAE, these findings highlight the need for targeted, population-specific prevention strategies, with particular emphasis on road safety and fall prevention in the domestic environment.

## Data Availability

The raw data supporting the conclusions of this article will be made available by the authors, without undue reservation.
